# SnO_2_-Based Porous Nanomaterials: Sol-Gel Formation and Gas-Sensing Application

**DOI:** 10.3390/gels9040283

**Published:** 2023-03-31

**Authors:** Irina Kononova, Vyacheslav Moshnikov, Pavel Kononov

**Affiliations:** 1 Department of Micro- and Nanoelectronics, Faculty of Electronics, Saint-Petersburg Electrotechnical University “LETI”, 5, pr. Popova, 197022 Saint-Petersburg, Russia; 2 Department of Descriptive Geometry and Graphics, Faculty of Basic and Human Sciences, Saint-Petersburg Mining University, 2, 21st Line, 199106 Saint-Petersburg, Russia

**Keywords:** sol–gel process, porous nanocomposites, tin dioxide, indium oxide, gas sensitivity

## Abstract

Porous nanocomposites using two (tin dioxide–silica dioxide) and three (tin dioxide–indium oxide-silica dioxide)-component systems for gas sensors were created with the sol–gel method. To understand some of the physical–chemical processes that occurred during the adsorption of gas molecules on the surface of the produced nanostructures, two models—the Langmuir model and the Brunauer–Emmett–Teller theory—were used to carry out calculations. The results of the phase analysis concerning the interaction between the components during the formation of the nanostructures were obtained through the use of X-ray diffraction, thermogravimetric analysis, the Brunauer–Emmett–Teller technique (to determine the surface areas), the method of partial pressure diagrams in a wide range of temperatures and pressures and the results of the measurement of the nanocomposites’ sensitivity. The analysis allowed us to find the optimal temperature for annealing nanocomposites. The introduction of a semiconductor additive into a two-component system based on tin and silica dioxides significantly increased the sensitivity of the nanostructured layers to reductional reagent gases.

## 1. Introduction

The introduction of nanotechnologies has allowed us to design completely new composite, constructive and functional nanomaterials [1,2,3,4] with considerably improved physical and chemical properties [5,6,7,8,9,10,11,12,13,14,15,16,17]. One of the fastest-growing directions in nanotechnologies concerns the development of functional porous materials based on metal oxide [18].

Functional, porous materials based on metal oxide can be obtained through the use of the sol–gel method [19,20], which deals with substances in their liquid state at the synthesis stage and homogenizes the initial components at the molecular level. Sol–gel technology [21] refers to economical and environmentally friendly technologies. Sol–gel thermodynamically nonequilibrium processes are widely used in the field of nanotechnologies [22,23,24,25]. At all stages of the sol–gel processes, diverse reactions that influence the composition and structure of the xerogel take place. It has been shown that the basis of sol–gel methods is the production of a homogeneous sol from the precursors and its conversion into a gel. The solvent in the gel is then removed from the gel structure and the remaining gel is dried. The properties of the dried gel significantly depend on the drying method. In other words, the “removing solvent method” is selected according to the application for which the gel is to be used [26,27,28].

As a rule in microelectronics, sol–gel technologies produce layers that have to meet smoothness and homogeneity requirements. When it comes to using gas-sensitive sensors, obtaining nanocomposite layers with controlled and replicable pore sizes is of high interest. In this case, nanocomposite materials have to contain an adhesion-improving phase and one or more phases of semiconductor n-type (conductivity) metal oxides to ensure gas sensitivity [29,30,31]. Metal oxide semiconductor compounds of n-type electrical conductivity, such as SnO_2_, ZnO, TiO_2_, WO_3_, Fe_2_O_3_, NiO and In_2_O_3_, are used as primary sensing elements in sensor devices. Of the listed materials, SnO_2_ is the most promising material for creating semiconductor gas sensors.

Tin oxide [32,33,34] as a semiconductor metal oxide has revealed great potential in the field of gas sensing due to its porous structure and reduced size. Especially for tin oxide and its composites, their inherent properties, such as high surface areas and unique semiconducting properties with tunable band gaps, make them compelling for sensing applications [35,36,37,38,39,40]. The electrical conductivity of tin dioxide is very sensitive to the state of surfaces in the region of elevated temperatures, at which redox reactions occur on the surface of the oxides. It is important to note that the high adsorption capacity, which is due to the presence of free electrons in the conduction band of the semiconductor, surface and oxygen vacancies, plays a significant role in chemisorption centres for atmospheric oxygen.

Film structures based on tin dioxide have high mechanical strength and chemical resistance. The destruction of films occurs only when they are exposed to HF or during prolonged processing in alkali.

Resistive gas sensors are considered promising candidates for gas detection, benefiting from their small size, ease of fabrication and operation convenience [41,42,43]. The way semiconductor gas sensors built onto percolating structures of metal oxide layers work is based on changes in the electrophysical properties during the adsorption of charged forms of oxygen, and the desorption of products of reactions between these layers and molecules of reductional gases [44,45,46].

The sol–gel method allows us to create porous materials with a large surface area, which can be used for gas sensors. Gas-sensitive porous nanomaterials can contain several types of pores simultaneously (macro–meso–microporous systems). It should be noted that, according to the IUPAC recommendation, three main types of pores are generally distinguished, i.e., micropores of sizes below 2 nm, mesopores in sizes ranging from 2 to 50 nm and macropores of sizes over 50 nm. This classification is based on differences in the basic mechanisms of sorption processes that take place in pores of different sizes.

In refs. [47,48], it is shown that the analytical capabilities of atomic force microscopy do not allow for the diagnosis of elements with sizes smaller than the locality of the method; therefore, the micropore system (less than 2 nm in size) cannot be detected. At the same time, the contribution of such pores to the total surface area can be determined by using the BET technique. To determine the surface area of the scanned areas using AFM, a technique based on the triangulation method was developed. The essence of the technique consists of the sequential approximation of the surface of the investigated area by a set of triangles, and then measuring their area. The area of the scanned area was calculated as the sum of the areas of 130,050 triangles with vertex coordinates corresponding to the coordinates of the vertices of 65,025 squares into which the AFM image was divided. It was found that the surface area of the porous nanocomposites calculated by processing atomic force images differed by 2–3 orders of magnitude from the surface area calculated according to the BET data. This indicated the existence of a micropore system of less than 10 nm, which was not diagnosed using AFM [47,48].

The purpose of this work was to create two types of sol–gel porous xerogels (two-component porous nanocomposites, SiO_2_–SnO_2_, and three-component porous nanocomposites, SiO_2_–SnO_2_–In_2_O_3_) to study the obtained samples with the use of different methods, to select the optimal annealing temperatures of three-component sol–gel systems (SiO_2_–SnO_2_–In_2_O_3_), to propose two sensor layer models with pores of different sizes and to create highly sensitive sensor layers.

## 2. Results and Discussion

The thermal treatment of nanocomposites in a SiO_2_–SnO_2_–In_2_O_3_ system can trigger some undesirable (in terms of the formation of nanostructured layers) chemical processes. That is why during the work, a phase analysis of the component interaction processes using the X-ray phase and thermogravimetric analysis (TGA) methods was conducted, as well as an analysis based on building a partial pressure diagram for a wide range of temperatures and pressures (since an X-ray phase analysis does not yield information about the X-ray amorphous phase of silica dioxide).

### 2.1. Thermodynamic Analysis of Phase Equilibriums in SiO_2_–SnO_2_–In_2_O_3_ System through the Construction of Partial Pressure Diagram

**Figure 1:**
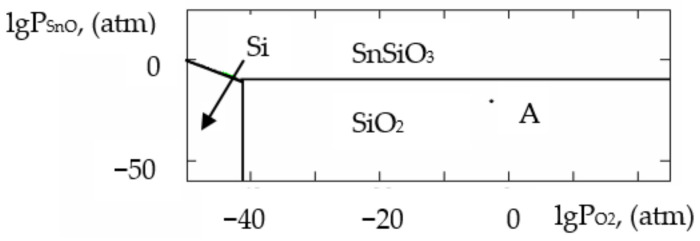
PPD in Sn–Si–O system at temperature T = 873 K within lgP_O2_–lgP_SnO_ coordinates.

**Figure 2:**
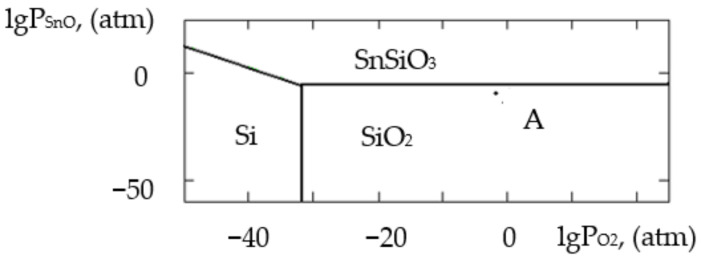
PPD in Sn–Si–O system at temperature T = 1073 K within lgP_O2_–lgP_SnO_ coordinates.

**Figure 3:**
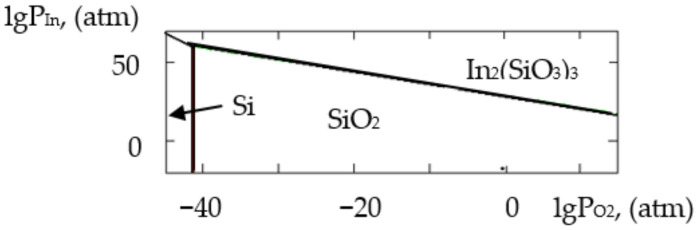
PPD in In–Si–O system at temperature T = 873 K within lgP_O2_–lgP_In_ coordinates.

**Figure 4:**
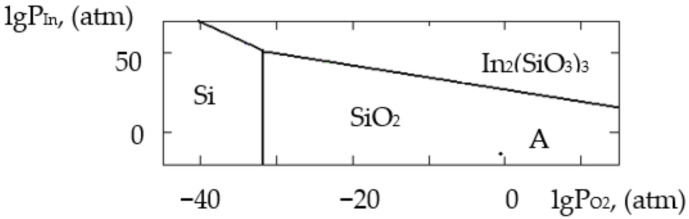
PPD in In–Si–O system at temperature T = 1073 K within lgP_O2_–lgP_In_ coordinates.

**Figure 5:**
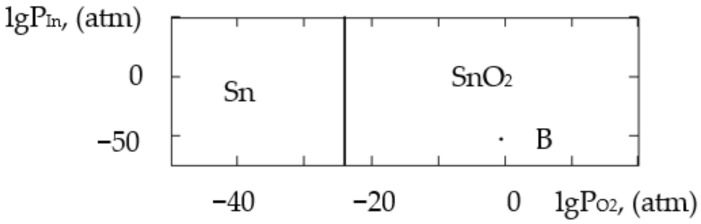
PPD in In–Sn–O system at temperature T = 873 K within lgP_O2_–lgP_In_.

**Figure 6:**
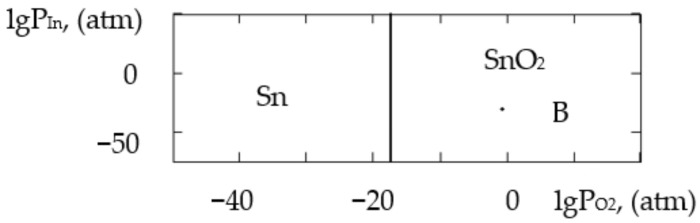
PPD in In–Sn–O system at temperature T = 1073 K within lgP_O2_–lgP_In_.

**Figure 7:**
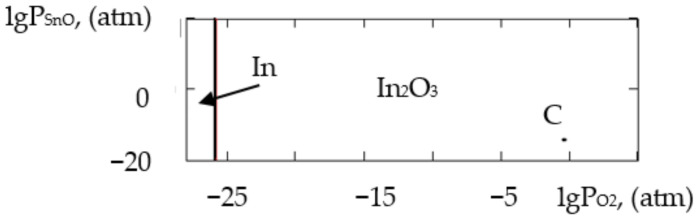
PPD in In–Sn–O system at temperature T = 873 K within lgP_O2_–lgP_SnO_.

**Figure 8:**
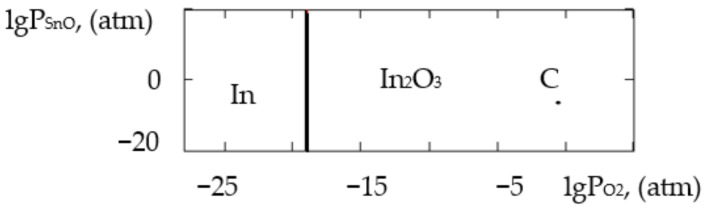
PPD in In–Sn–O system at temperature T = 1073 K within lgP_O2_–lgP_SnO_.

The construction of a partial pressure diagram (PPD) was based on an analysis of the conditions of an equilibrium in a heterogeneous chemical reaction in the presence of gas-like substances. The PPD showed areas of resistance or a condensed phase depending on the composition of the gas phase, as well as the isothermal cross-sections of the PPD of three-component systems, including a gas phase consisting of two gases. As a result of the X-ray phase analysis, the condensed phases of such a system were either in a solid or liquid state and did not form solid solutions. Phase equilibriums in a Sn–Si–In–O system can be represented in a geometrical way using an N-dimension space, with N being greater than three, but in order to simplify the calculations and provide it in a more graphic way, the system was divided into three subsystems. It is known [49] that the vapour pressure of Si, SiO and SiO_2_ above the solid phase of SiO_2_ is considerably lower than the pressure of SnO vapours above solid SnO_2_. This way, in the gas phase, except for oxygen, gaseous SnO was prevalent over composite materials consisting of SnO_2_ and SiO_2_, that is why the PPD for the Sn–Si–O subsystem was built within the lgP_O2_–lgP_SnO_ coordinates (the half-logarithmic temperature dependence of SnO (lg P_SnO_ = f(T)) vapour pressures is shown in Table 1). Since all the processes took place in air (at atmospheric pressure), it was assumed that Po_2_ = 0.21 atm. The reagents were chosen and the chemical interaction equation system was built in such a way that at least one gaseous component was part of the equilibrium (Table 2). It was known [49] that the pressure of InO and In_2_O vapours above the solid phase of In_2_O_3_ and Si, SiO and SiO_2_ above the solid phase of SiO_2_ was significantly lower than the pressure of In vapours above solid In_2_O_3_. This way, in the gas phase, gaseous In was prevalent over composite materials consisting of In_2_O_3_ and SiO_2_, which is why the PPD for the In–Si–O subsystem was built within the lgP_O2_–lgP_In_ coordinates (the half-logarithmic temperature dependence lg P_In_ = f(T) is shown in Table 3). The choice of reagents and the composition of the equation system of their interactions in the In–Si–O subsystem are shown it Table 4. The PPD for the In–Sn–O was built within the lgP_O2_–lgP_In_ and lgP _O2_–lgP_SnO_ coordinates. The choice of reagents and the construction of the equation system of their interactions in the In–Sn–O are shown in Table 5. The calculations of the reaction constants were performed using the standard thermodynamic procedure with the following ratios:

(1) $$\begin{array}{l} \Delta G_{T}^{0}=\Delta H_{298}^{0}+\int_{298}^{T}\Delta C_{p}^{0}dT-T\left(\Delta S_{298}^{0}+\int_{298}^{T}\frac{\Delta C_{p}^{0}}{T}dT\right)\\ \Delta G_{T}^{0}=-RT\ln K_{p}, \end{array}$$

where $\Delta H_{298}^{0}$ is the change in the standard reaction enthalpy, $\Delta S_{298}^{0}$ is the change in the standard reaction entropy, $\Delta C_{p}^{0}$ in the change in the heat capacity (the thermodynamic characteristics in the Sn–Si–In–O system are shown in Table 6), $\Delta G_{T}^{0}$ is the change in free Gibbs energy, $K_{p}$ is the equilibrium constant and T is the temperature. The value of $\Delta C_{p}^{0}$ for the monovariant equilibriums was small, which is why the calculations were conducted using the Ulich approximation: $\Delta C_{p}^{0}=\Delta C_{p298}^{0}$. The results of the calculations of the equilibrium constants of the chemical interactions are shown in Table 6 and in Figure 1, Figure 2, Figure 3, Figure 4, Figure 5, Figure 6, Figure 7 and Figure 8.

The calculations of the pressures of the saturated SnO and In vapours are shown in Table 6. The vapours of SnO and In in the Sn–Si–O, In–Si–O and In–Sn–O systems were only present in the gas phase at partial pressures lower than those in Table 6; if those pressures were reached, the gaseous components would condense. From the PPD in the Sn–Si–In–O system, it followed that under the conditions of the experiment (at the atmospheric pressure and studied temperature), the next were stable:

- The phase of amorphous SiO_2_ (point A, Figure 1, Figure 2, Figure 3 and Figure 4);
- The phase of crystalline SnO_2_ (point B, Figure 5 and Figure 6);
- The phase of crystalline In_2_O_3_ (point C, Figure 7 and Figure 8).

### 2.2. Studying the Xerogel Powder Samples Using XRD, TGA and BET

**Figure 9:**
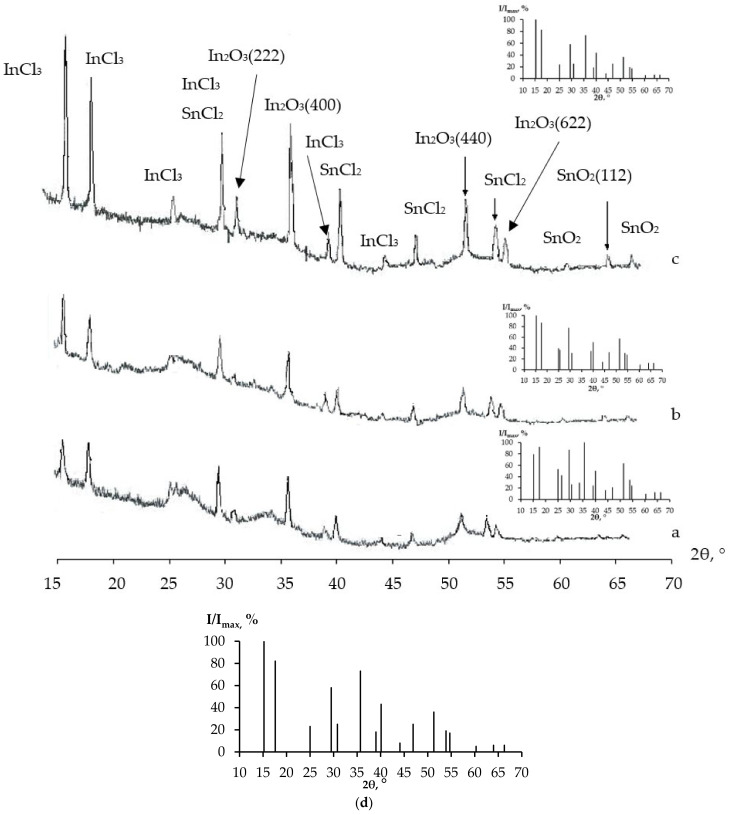

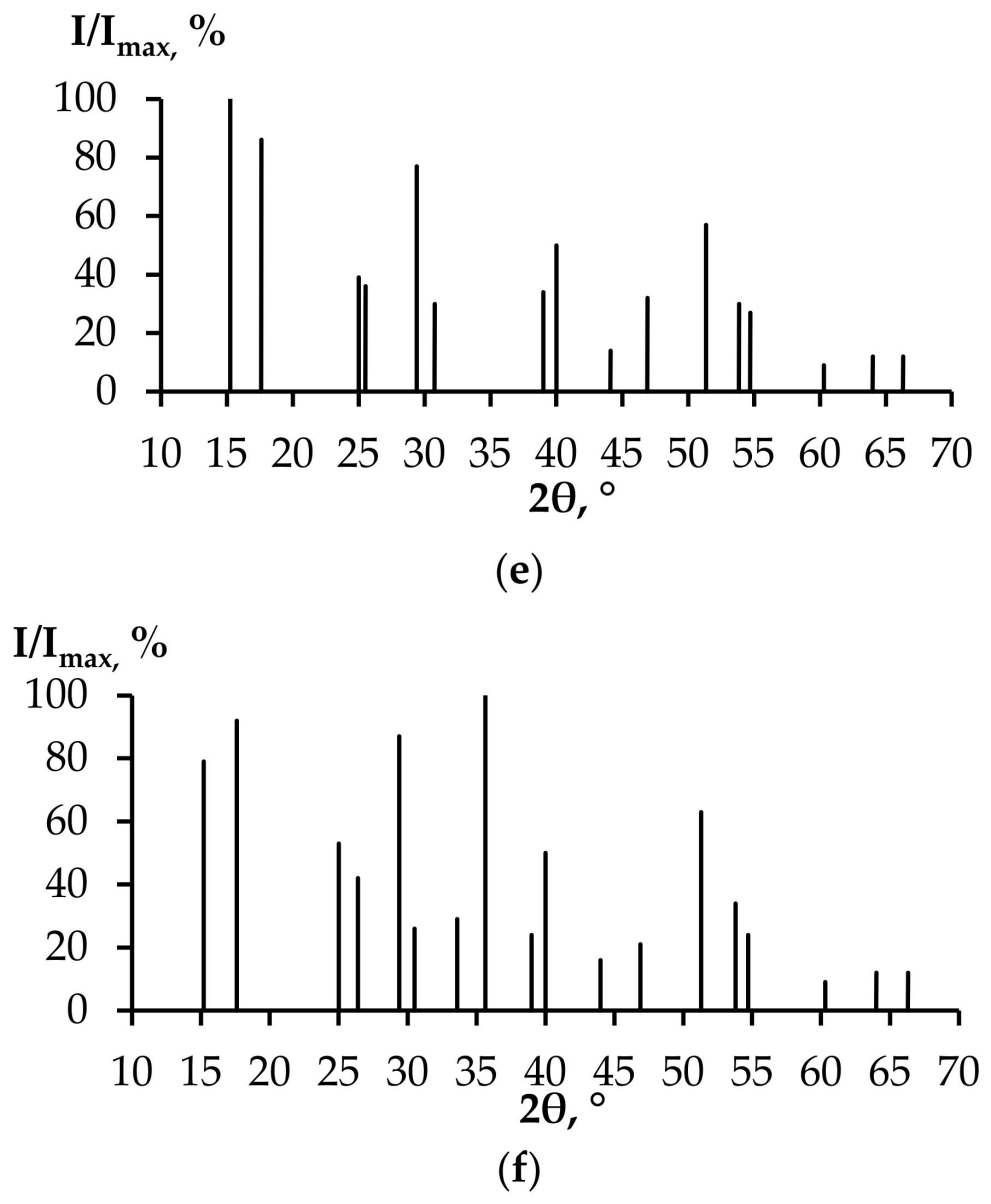
XRD patterns (after thermal treatment at 300 °C) showing the intensity of diffracted X-rays in planes as a function of 2θ for three samples: (**a**,**f**) 10SiO_2_–85SnO_2_–5In_2_O_3_; (**b**,**e**) 10SiO_2_–80SnO_2_–10In_2_O_3_; (**c**,**d**) 10SiO_2_–75SnO_2_–15In_2_O_3_ (the Miller indices (hkl) are also shown beside the peaks).

**Figure 10:**
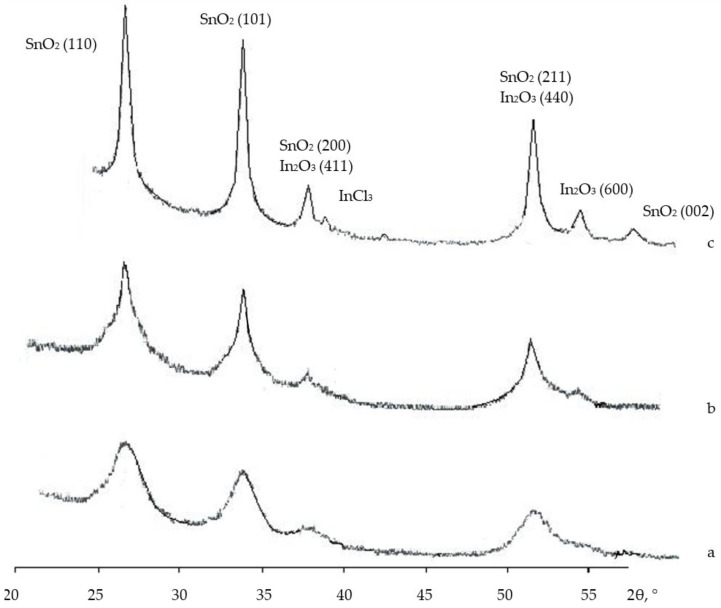

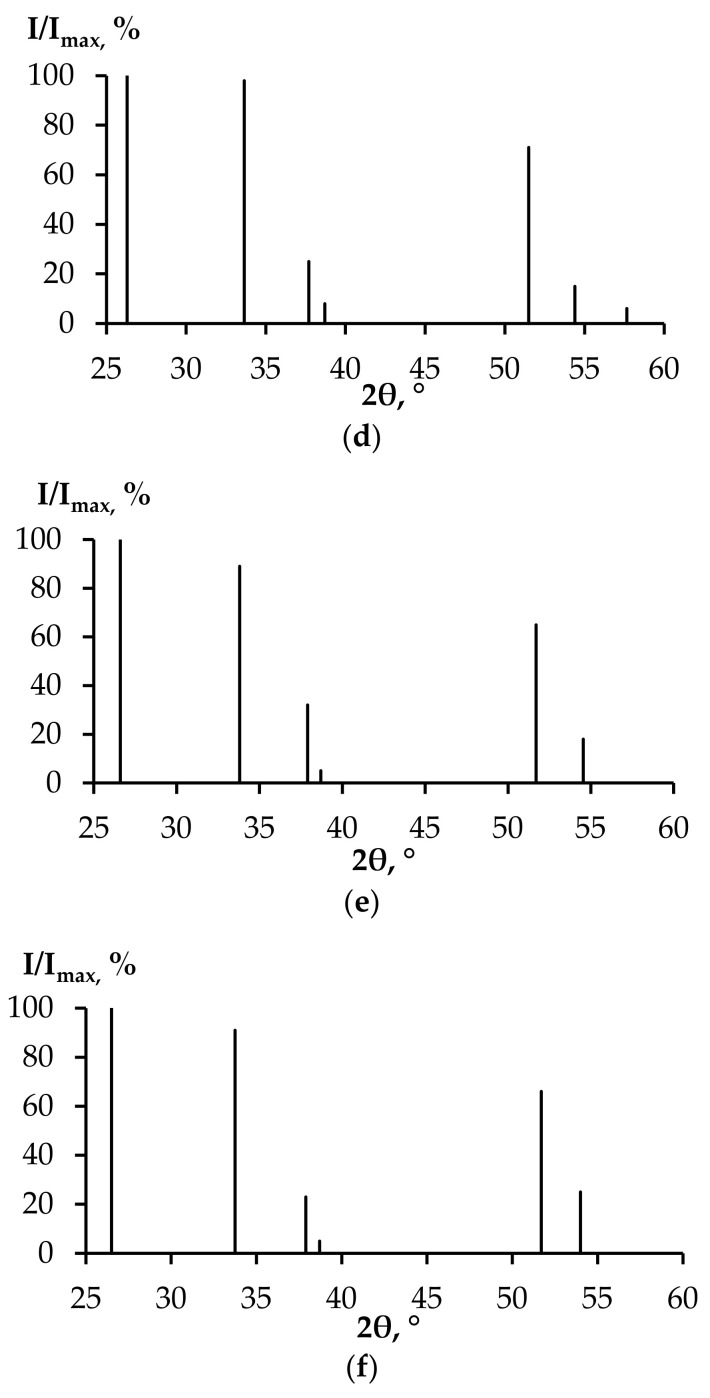
XRD patterns (after thermal treatment at 600 °C) showing the intensity of diffracted X-rays in planes as a function of 2θ for three samples: (**a**,**f**) 10SiO_2_–85SnO_2_–5In_2_O_3_; (**b**,**e**) 10SiO_2_–80SnO_2_–10In_2_O_3_; (**c**,**d**) 10SiO_2_–75SnO_2_–15In_2_O_3_ (the Miller indices (hkl) are also shown beside the peaks).

The features of nanocomposites were studied using gel powders produced from the initial gel. The results of the study conducted using the X-ray phase analysis method in SiO_2_, SnO_2_ and In_2_O_3_ systems are shown in Figure 9 and Figure 10.

**Figure 11:**
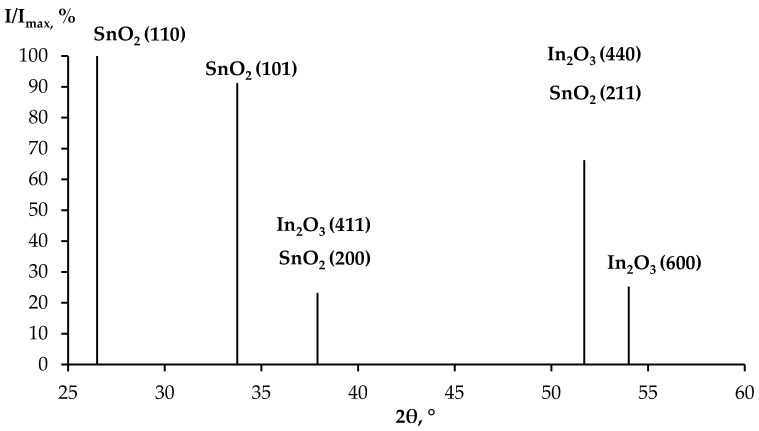
Typical XRD pattern (after thermal treatment at 700 °C).

An analysis of the X-ray phase analysis data for the nanocomposite powders (10SiO_2_–85SnO_2_–5In_2_O_3_, 10SiO_2_–80SnO_2_–10In_2_O_3_ and 10SiO_2_–80SnO_2_–10In_2_O_3_ (mas. %) showed that after a thermal treatment at 300 °C (Figure 9), the peaks of the SnCl_2_ and InCl_3_ phases occurred, as well as peaks reflecting the initial crystallization of the SnO_2_ and In_2_O_3_ phases. As the concentration of indium salts in the sample grew, the general appearance of the diffractograms stayed the same, but a widening of peaks in the InCl_3_ phase alongside a decrease in their intensity was noticed, as well as a more pronounced crystallization of the SnO_2_ phase. It may be assumed that chloride anions were captured by positively charged ions of indium. During this, the released tin ions actively interacted with oxygen atoms, as a result of which SnO_2_ crystallites were formed. After a treatment at 600 °C, the SnCl_2_ phase peaks disappeared completely, but the InCl_3_ phase peaks remained, and well-formed SnO_2_ phase peaks appeared (SnO_2_ (110), SnO_2_ (101), SnO_2_ (200), SnO_2_ (211) and SnO_2_ (002)), while the intensity of the In_2_O_3_ phase reflexes decreased (In_2_O_3_ (411), In_2_O_3_ (440) and In_2_O_3_ (600)). At the same time, as the concentration of indium salts grew, the general appearance of the diffractograms remained the same, but the SnO_2_ phase peaks widened, even in the small angle section, which indicated that the size of the SnO_2_ crystallites decreased. After a treatment at 700 °C, only the SnO_2_ and In_2_O_3_ phase peaks remained (Figure 11). The size of the crystallites was estimated with the Scherrer equation, and was equal to 17–37 nm.

**Figure 12:**
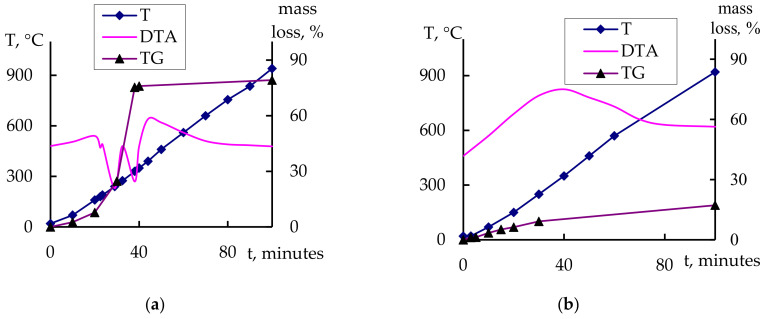
TGA curves: (**a**) after heat treatment at 100 °C for 50 h; (**b**) after heat treatment at 100 °C for 50 h and 600 °C for 1 h (10SiO_2_–75SnO_2_–15In_2_O_3_).

According to the results of the TGA, at 100 °C (Figure 12a), endothermic effects occurred associated with a mass loss of 79% and the simultaneous crystallization of the material. This was confirmed using the XRD data; already at 300 °C, the crystallization of the SnO_2_ phase could be observed. The thermogravigram at 600 °C (Figure 12b) reflected a mass loss of 17% against the background of an exoeffect, indicating the continued crystallization of SnO_2_ and In_2_O_3_.

**Figure 13:**
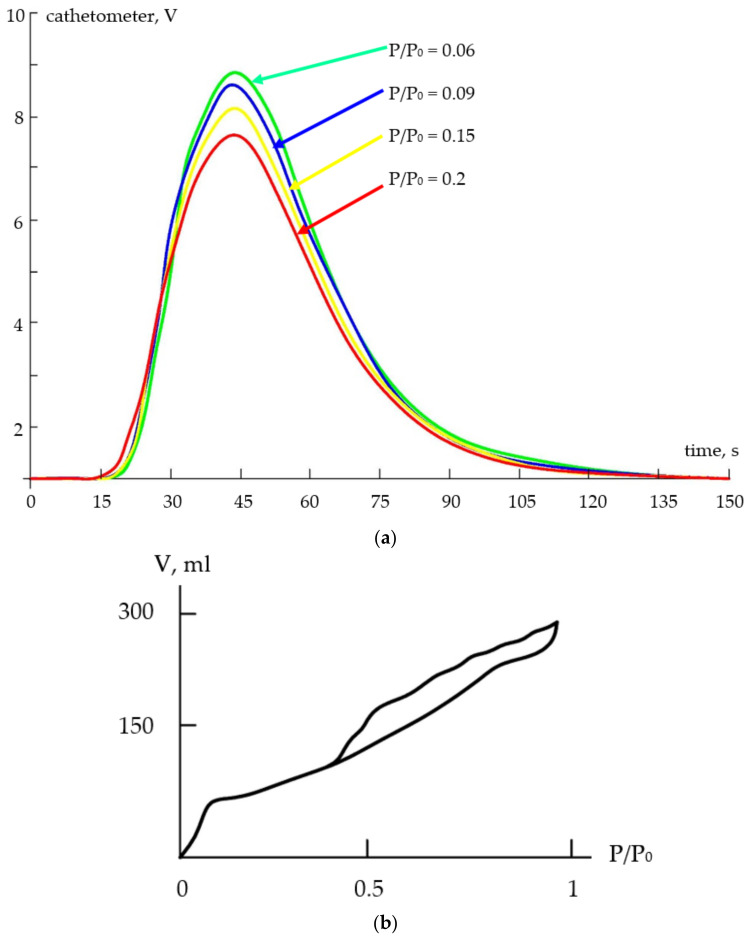
(**a**) Signals received from thermal conductivity meter formed during adsorption analysis; (**b**) a typical adsorption isotherm.

The specific surface areas of the nanocomposites were studied. A thermal conductivity sensor was used as a detector of the composition of the gas mixture, the output signal of which was a desorption peak (Figure 13) and the area of which was directly proportional to the volume of the desorbed gas. According to the results of the BET method, it was found that the specific surface area of the two-component system (tin dioxide–silica dioxide) was 80–100 m^2^/g. The surface area for the three-component system (tin dioxide–indium oxide–silica dioxide) was several times larger than the component in the two-component system.

### 2.3. Studying the Morphology of the Xerogel Films

**Figure 14:**
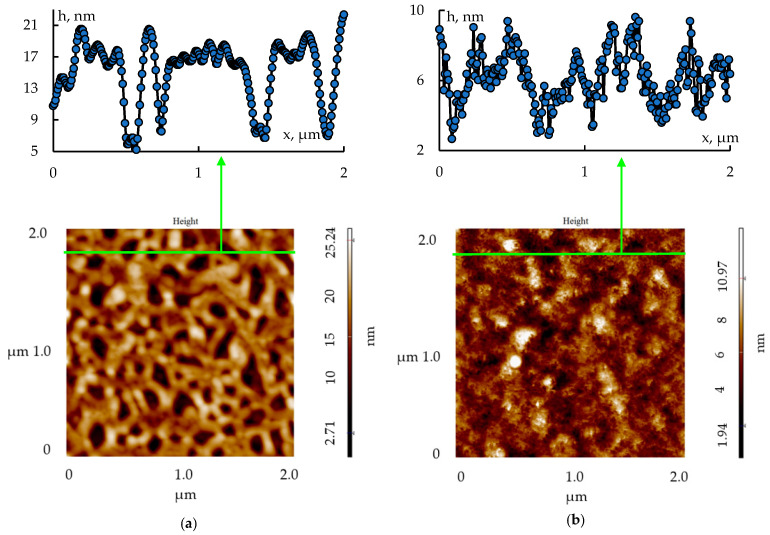
Typical atomic force microscopy images (image size 2 μm × 2 μm) of porous nanocomposites and their profiles (along the green lines): (**a**) a two-component system, SiO_2_–SnO_2_; (**b**) a three-component system, SiO_2_–SnO_2_–In_2_O_3_.

The morphology of the porous nanocomposites of two- and three-component systems (tin dioxide–silica dioxide and tin dioxide–indium oxide–silica dioxide) synthesized from sols were studied with the use of AFM. Typical atomic force microscopy images of porous nanocomposites are shown in Figure 14. It was shown that when indium oxide was introduced into a two-component structure, the pore sizes significantly decreased from 60–170 nm to 10 nm.

### 2.4. Studying the Electrophysical Properties of Film Nanocomposites

**Figure 15:**
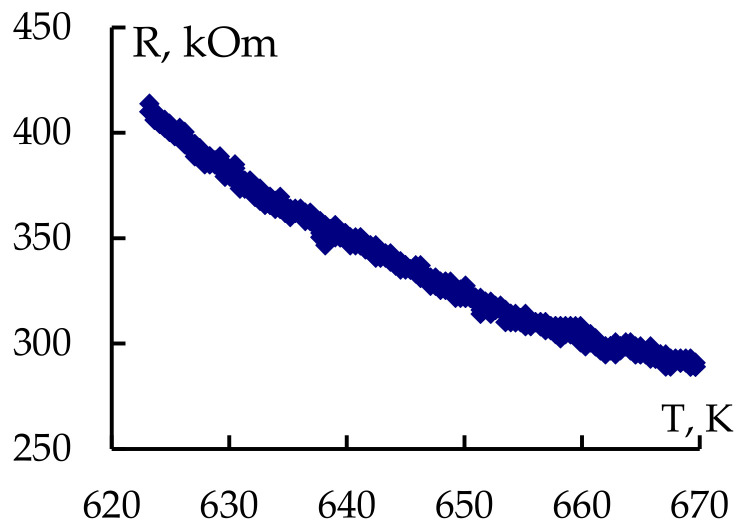
Typical image of temperature dependency of net-like sample produced using sol–gel method.

When studying the electrophysical properties, it was found that the resistance of structures decreased as the temperature rose, according to the exponential law:

(2) $$R=R_{0}\exp\left(\frac{E_{A}}{kT}\right).$$

where *R* is the resistance of the sample, $R_{0}$ is the typical value for this sample, k = 8.615·10^−5^ eV/K is the Boltzmann constant, *T* is the temperature and $E_{A}$ is the activation energy, which confirmed the semiconductor characteristic of the conductivity of the metal oxide structures. Using temperature dependencies (in the range of 300–400 °C, Figure 15), the activation energies of the conductivity of semiconductor nanocomposite materials were calculated (which were found to be (0.24–0.46 eV), as well as the temperature resistance coefficients equal to the relative change of the resistance when the temperature changed by 1 K, and was equal to:

(3) $$\alpha_{R}=\frac{1}{R}\frac{\partial R}{\partial T}=-(6÷11)\cdot 10^{-3}K^{-1}$$

It must be noted that the experimentally found activation energy could have been the so called ‘seeming’ activation energy differing from the thermal activation of charge carriers by the value of the thermal effect accompanying the process of oxygen adsorption on the surface of the semiconductor nanocomposites and connected with the existence of a potential barrier to the flow of electricity existing on the grain boundaries.

**Figure 16:**
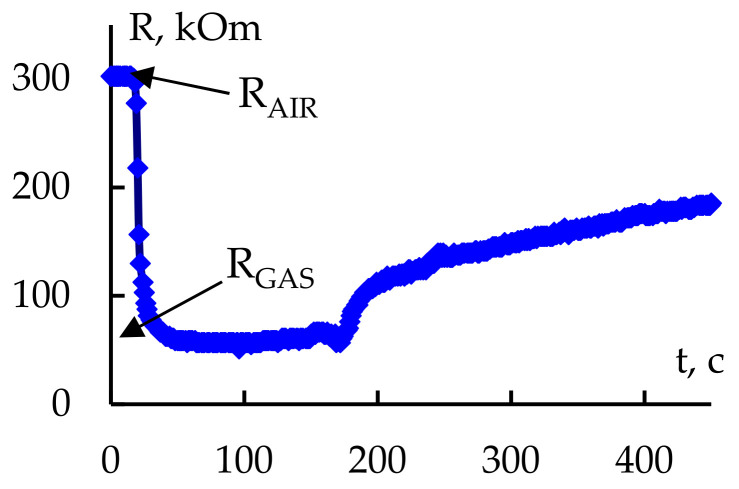
Typical temporal dependency of resistance of net-like nanocomposite during and just after introduction of reagent gas.

Figure 16 shows the typical change in the resistance of the sample under the impulse of reagent gas and the further change in the resistance during reduction. The research carried out to determine the sensitivity to acetone and ethanol fumes showed that the reduction time after the impulse was 2–40 min, depending on the detection temperature and sample structure. It was established that a temporary resistance reduction in semiconductor nanocomposites after the impulse of a reagent gas could be performed by sending a thermal (600 °C) impulse for 30 s; in this case, the sensitivity to reagent gases under the same detection conditions could increase 1.5–4 times due to the OH desorption from the surface and higher R_AIR_.

### 2.5. Analysis of Adsorption Processes on Nanocomposite Surfaces

When studying the resistance patterns of nanocomposites based on tin and silica dioxides in a constant electric field in an air environment in the presence of reductional fumes of ethanol and acetone, it was found that as the temperature increased, the reaction time decreased for some samples, while staying the same for others. To interpret the results, software compatible with the LabVIEW environment was developed using simplified traditional models of monomolecular adsorption on a homogeneous surface, which was produced without taking the interaction between adsorbents into consideration (the Langmuir model), as well as those of polymolecular adsorption based on monomolecular adsorption and taking ‘vertical’ intermolecular adsorbent interactions into account (the BET model).

Within the Langmuir model, the following assumptions were used: adsorption happened in separate adsorption centres, each of which could only retain one molecule of gas; the surface was energy-homogeneous, i.e., it only contained adsorption centres with the same adsorption heat bond energy between particular molecules of adsorbed gas; adsorbed molecules did not interact, and the strength of the bond between the molecule and the centre did not depend on the filling of adjacent centres; the number of adsorption centres on the surface was constant and did not depend on external conditions; each adsorption centre could only bond to the given molecule in one particular way; the bond energy was constant during the whole life span of the molecule in the adsorption centre.

Within the BET model, it was assumed that each particle of the first monolayer was the adsorption centre for the second layer, etc.; in all layers, except the first, the adsorption heat was equal to the condensation heat; in all layers, except the first, the condensation and evaporation conditions were the same; at a pressure equal to the pressure of the saturated vapours, adsorbed molecules condensed on the surface as a liquid, i.e., the number of layers became infinite.

This way, within the Langmuir model in the LabVIEW environment, the calculations were reduced to analytically solving a first-order linear partial differential equation:

(4) $$\frac{\partial \theta (t)}{\partial t}+(K_{ADS}+K_{DES})\cdot \theta (t)=K_{ADS}$$

which looked like this:

(5) $$\theta (t)=\exp(-\left\{K_{ADS}+K_{DES}\right\}\cdot t)\cdot K_{ADS}\cdot \left[\frac{1}{K_{ADS}+K_{DES}}\cdot \exp\left(\left\{K_{ADS}+K_{DES}\right\}\cdot t\right)+C\right]$$

where $C=-\frac{1}{K_{ADS}+K_{DES}}$, $K_{ADS}=\frac{p}{N_{C}\sqrt{2\pi \cdot M\cdot K_{B}\cdot T}}\cdot \exp\left(-\frac{E_{ADS}}{K_{B}T}\right)$ is the adsorption coefficient, $K_{DES}=\nu \cdot \exp\left(-\frac{E_{DES}}{K_{B}T}\right)$ is the desorption coefficient, $E_{ADS}$ and $E_{DES}$ are the adsorption and desorption activation energies, T is the temperature, $K_{B}$ is the Boltzmann constant, ν is the frequency of attempts to penetrate the desorption barrier, M is the mass of the adsorbed molecule, $N_{C}$ is the total concentration of occupied and empty spaces (concentration of adsorption centres on the surface), p is the steam pressure, *θ(t)* is the degree of the filling of the surface with adsorbed molecules and *t* is the filling time with adsorbed molecules.

That is why within the BET model in the LabVIEW environment, the calculations were reduced to solving a first-order partial differential equation using the Euler method, in which the right half was a cubic equation related to the degree of the filling of the surface with single complexes at a particular moment of time:

(6) $$\begin{array}{l} \frac{\partial \theta^{1}(t)}{\partial t}=-K_{ADS}\cdot {\left(\frac{p}{p_{S}}\right)}^{2}\cdot {\left\{\theta^{1}(t)\right\}}^{3}+\\ +\left[K_{ADS}\cdot {\left(\frac{p}{p_{S}}\right)}^{2}-2\cdot \left(1-\frac{p}{p_{S}}\right)\cdot \frac{p}{p_{S}}\cdot K_{ADS}-\frac{K_{DES}}{1-\frac{p}{p_{S}}}\cdot \frac{p}{p_{S}}\right]{\left\{\theta^{1}(t)\right\}}^{2}+\\ +\left[K_{ADS}\cdot 2\cdot \left(1-\frac{p}{p_{S}}\right)\cdot \frac{p}{p_{S}}-{\left(1-\frac{p}{p_{S}}\right)}^{2}\cdot K_{DES}-K_{DES}\right]\cdot \theta^{1}(t)+\\ +K_{ADS}\cdot {\left(1-\frac{p}{p_{S}}\right)}^{2} \end{array}$$

where $p_{S}$ is the pressure of the saturated gas and $\theta^{1}(t)$ is the degree of the surface filling with single complexes.

**Figure 17:**
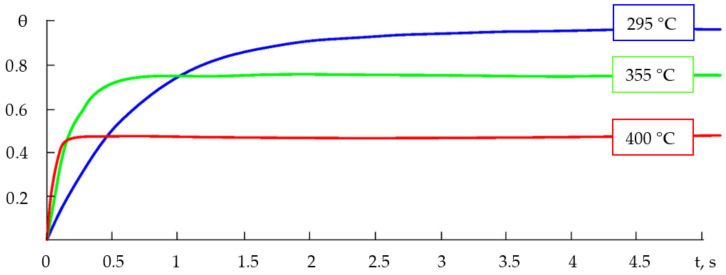
Typical dependencies of degree of filling on time (at different temperatures within Langmuir model).

Within the used models, the maximum possible degree of the filling of adsorption centres and the speed of the filling were affected by such parameters as the pressure, temperature, mass of adsorbed molecules and the energies of adsorption and desorption activation (Figure 17).

**Figure 18:**
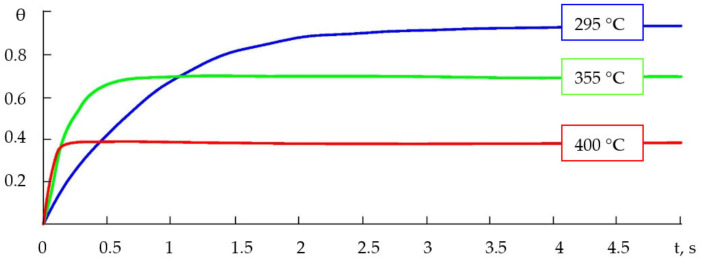
Typical dependencies of degree of filling on time (at different temperatures within BET model).

Based on the results of calculations in the LabVIEW environment, it was found that raising the temperature in one range resulted in a decrease in the amount of time needed to complete the adsorption processes (finding an equilibrium), while, in another temperature range (relatively higher), it led to a decrease in the degree of the filling of the surface with adsorbed particles and the filling time. With the other parameters of the system being equal, both the Langmuir and BET systems (Figure 17 and Figure 18) produced similar correlations between the degree of filling and time, and differed mostly in the filling time; then, since the calculations within the monomolecular model were more trivial, the further analysis of the features of the dependencies of the degree of gas molecule filling was conducted within the Langmuir model.

**Figure 19:**
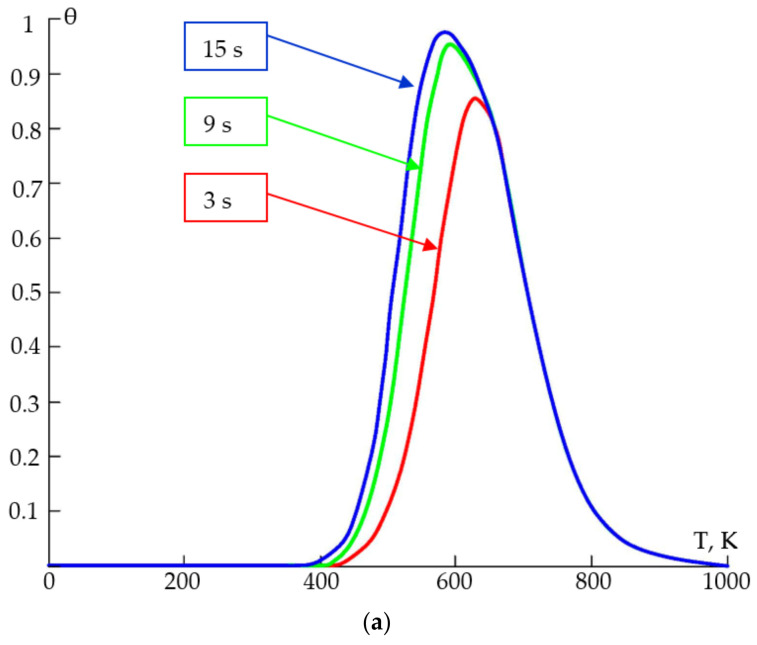

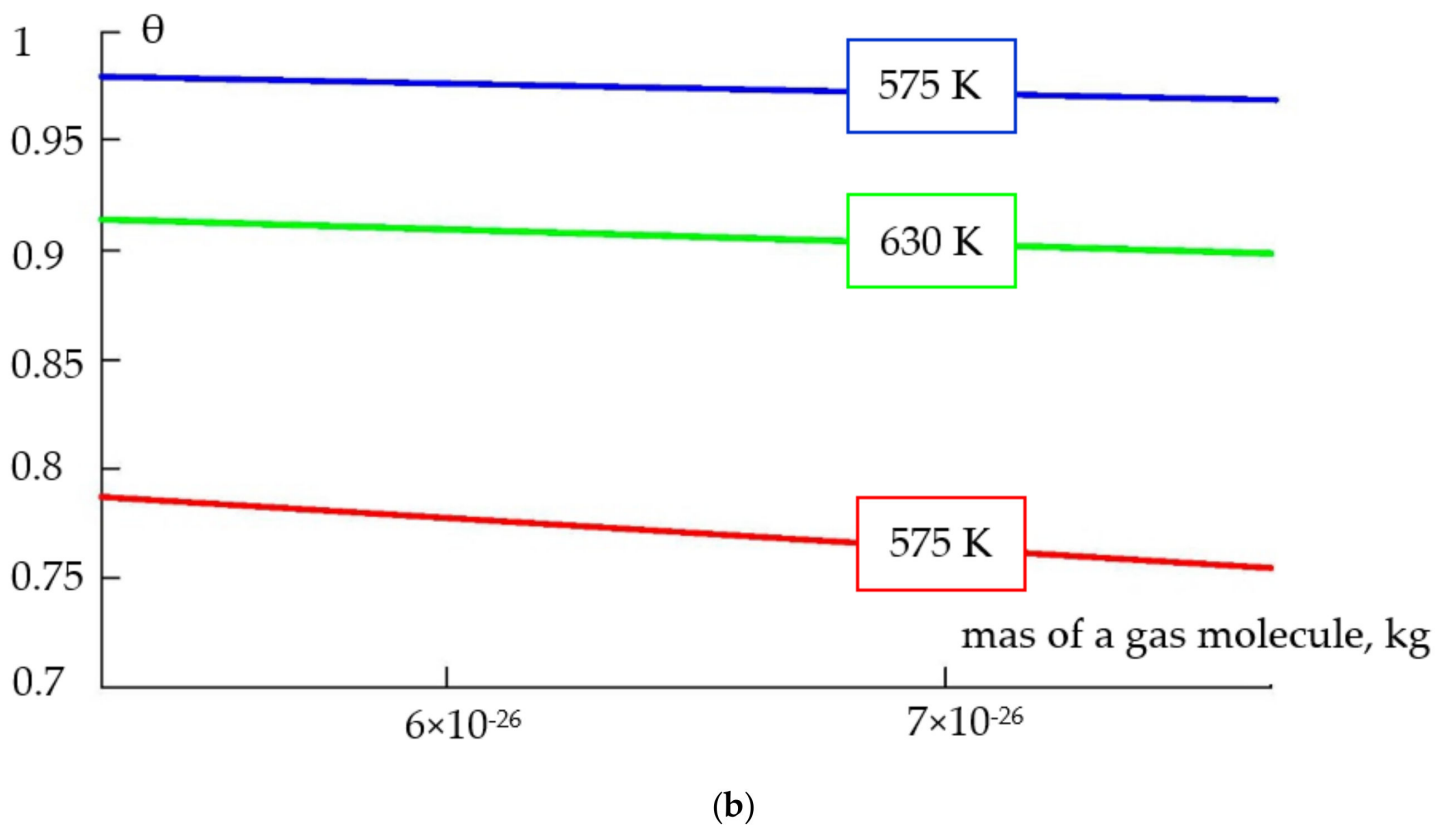
The dependence of degree of filling on temperature at different filling times (**a**); mass of a gas molecule at different temperatures (**b**).

Comparing the experimental gas sensitivity data to the results of computer modelling showed the existence of an optimal temperature range for the detection process, outside which the degree of the surface filling in a particular adsorber–adsorbent system decreased, and which was found to be between 300 and 400 °C (573–673 K) (Figure 19a). At temperatures close to room temperature, the adsorption process would proceed quite slowly; for example, at room temperature (293 K), the adsorption of ethanol vapours could take up to 3 h, in which case, the filling degree would be θ = 0.2 (within the Langmuir and BET models). It was shown that the filling degree depended on the mass of the molecule—the heavier the molecule, the lower the degree (Figure 19b).

It was also found that for samples characterized by a decrease in sensitivity to reductional gases and reaction time, the typical ratio of ‘effective’ activation temperatures of adsorption and desorption was 2.5 to 1, while for porous nanocomposites characterized by the same sensitivity to reductional gases and an insignificant change in reaction time when the temperature rose, the ratio was more than 3.5 to 1.

To increase the sensitivity S = (R_AIR_ − R_GAS_)/R_GAS_ of the adsorption semiconductor sensory layers, it was considered possible to create a relatively high resistance in air R_AIR_ and a relatively low resistance of structures in the presence of a reagent gas R_GAS_. This was achieved through the deliberate introduction of indium oxide into a system based on tin and silica dioxides (the introduction of a nonorganic salt of indium at the stage of the preparation of the polymeric sol–solutions). Measurements of the gas sensitivity of nanocomposites based on tin and silica dioxides and indium oxide were carried out using reductional ethanol and acetone vapours. An analysis of these measurements using PPD, an X-ray phase analysis and TGA data resulted in the establishment of the optimal temperature of nanocomposite annealing (700 °C), below which the sensitivity to reductional gases decreased. It must be noted that if the nanocomposites were annealed at a temperature below the optimal one, an anomalous increase in resistance in the presence of reagent gases was observed, which might be explained by the presence of indium chloride in the nanostructures of the salt, which could have significantly reduced the resistance in the samples. The presence of this phase, especially at high temperatures of gas detection, could have caused an anomalous curve in the gas sensitivity graph.

According to the gas sensitivity measurements, it was established that the deliberate introduction of a semiconductor additive of indium oxide into a two-component system, SiO_2_–SnO_2_, increased the sensitivity of the nanostructured layers to reductional reagent gases tenfold (S = 2–30 (for the nanocomposites of a two-component system, SiO_2_–SnO_2_) to S = 100–250 (for the nanocomposites of a three-component system, SiO_2_–SnO_2_–In_2_O_3_)), which was caused by a relatively high resistance of the samples in air R_AIR_.

## 3. Conclusions

**Figure 20:**
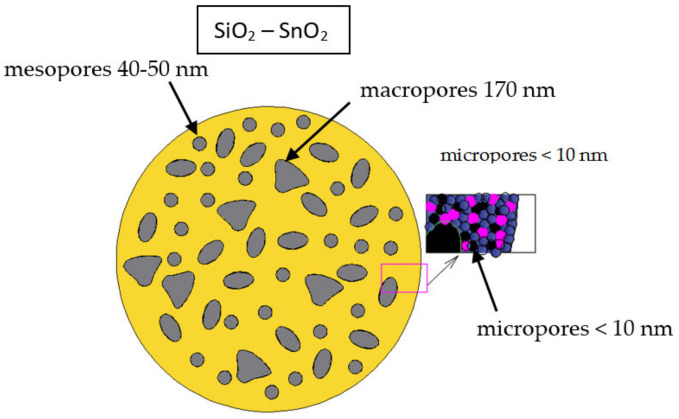
Hierarchical model of the formation of two-component porous nanocomposites.

**Figure 21:**
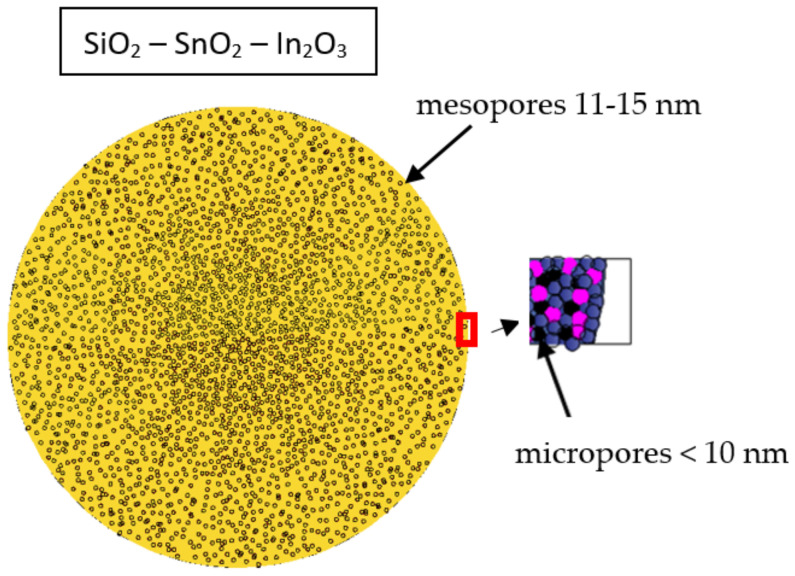
Hierarchical model of the formation of three-component porous nanocomposites.

The analysis of gas sensitivity measurements based on PPD and X-ray phase analysis data allowed us to find the optimal temperature for annealing nanocomposites of a three-component system, SiO_2_–SnO_2_–In_2_O_3_ (700 °C). Based on complex studies of AFM, the BET technique and on previous research [47,48], a hierarchical model of the formation of two-component and three-component porous nanocomposites was proposed, presented in Figure 20 and Figure 21 (in the inset, the semiconductor and dielectric grains of nanocomposites are indicated in pink and blue), accordingly. According to the AFM data, it was found that two-component porous nanocomposites, SiO_2_–SnO_2_, were macro–meso–microporous (macropore sizes of 170–180 nm and mesopore sizes of 40–50 nm) and three-component porous nanocomposites, SiO_2_–SnO_2_–In_2_O_3_, were meso–microporous (mesopore sizes of 11–15 nm). According to the gas sensitivity measurements, it was established that the deliberate introduction of a semiconductor additive of indium oxide into a two-component system, SiO_2_–SnO_2_, increased the sensitivity of the nanostructured layers to reductional reagent gases by tenfold.

Based on temperature dependencies (within the temperature range 300–400 °C), the activation energies of sample conductivity were found to be 0.24 ÷ 046 eV, and the thermal resistance coefficients were found to be $\alpha_{R}=\frac{1}{R}\frac{\partial R}{\partial T}=-(6÷11)\cdot 10^{-3}K^{-1}$.

A complex analysis of experimental data related to gas sensitivity and the calculation results within the Langmuir and BET models in the integrated LabVIEW system allowed us to establish the optimal temperature range for the detection process, outside of which the filling degree of the surface for a particular adsorber–adsorbent system decreased, which was shown to be between 300 and 400 °C.

It was shown that for samples characterized by a decrease in sensitivity to reductional gases and in reaction time when the temperature rose within the 250–400 °C range, the ratio of the activation energy for adsorption and desorption was 2.5 to 1, while for porous nanocomposites characterized by the same sensitivity to reductional gases and an insignificant change in reaction time under the same conditions, the ratio was 3.5 to 1.

It was experimentally revealed that the hydrolysis and polycondensation reactions of Si(OC_2_H_5_)_4_ in the presence of not one, but two inorganic metal salts (SnCl_2_ · 2H_2_O and In_2_(SO_4_)_3_), at the sol preparation stage significantly increased the sensitivity of the porous nanomaterials to reducing gas vapours under the condition of annealing film xerogels at an optimal temperature of 700 °C. Therefore, the addition of indium oxide into a two-component system, SiO_2_–SnO_2_, increased the sensitivity of the nanostructured layers to reductional reagent gases at a detection temperature in the range from 300 to 400 °C tenfold (S = 2–30 (for the nanocomposites of a two-component macro–meso–microporous system, SiO_2_–SnO_2_) to S = 100–250 (for the nanocomposites of a three-component meso–microporous system, SiO_2_–SnO_2_–In_2_O_3_)), which was caused by the samples’ relatively high resistance in air $R_{AIR}$ of the latter.

## 4. Materials and Methods

Tetraethyl orthosilicate Si(OC_2_H_5_)_4_, tin (II) chloride and indium (III) sulphate were used as the precursors. The initial components used to create the sols were easily hydrolysed chemicals that, when allowed to interact with water, formed polymolecules or polysolvated groups. Tetraethyl orthosilicate hydrolyse and polycondensation reactions were conducted in the presence of metal oxide sources (inorganic salts, namely, tin (II) chloride and indium (III) sulphate). H_2_SO_4_ and HCl were added at the stage of dissolving SnCl_2_ · 2H_2_O and In_2_(SO_4_)_3_.

Materials based on SiO_2_, SnO_2_ and In_2_O_3_ and produced through sol–gel synthesis were investigated in this study. The source of SiO_2_ was Si(OC_2_H_5_)_4_ (tetraethoxysilane, TU2637-187-44493179-2014, Moscow, Russia), the source of SnO_2_ was SnCl_2_·_2_H_2_O (GOST 36-78, Saint-Petersburg, Russia) and the source of In_2_O_3_ was In_2_(SO_4_)_3_ (TU 6-09-3756-80, Moscow, Russia).

Inorganic salts of tin and indium were dissolved in butyl alcohol until completely dissolved (as a result, transparent solutions were obtained). Then, tetraethyl orthosilicate was added. Two types (xerogel films and xerogel powders) of porous nanocomposites from sols were created.

A water solution of ammonia was used to turn the sols into gels (for creating xerogel powders). The produced wet gel was dried out at a temperature of 100 °C for 50 h and later kept at constant temperatures of 300 °C and 700 °C. To study the xerogel powder samples using X-ray diffraction (XRD, DRON 3.0, Burevestnik, Saint-Petersburg, Russia) and thermal gravimetric analysis (TGA, laboratory installation, St. Petersburg, Russia), the BET (Brunauer–Emmett–Teller) technique was used to determine the surface areas of the xerogel powder samples. The characteristics of the xerogel powders were studied using a gas-adsorbent Sorbi-series analyser (Meta, CJSC, Novosibirsk) by comparing the volumes of the adsorbent gas (nitrogen) adsorbed by the sample, with samples (specific surface area of 106 m^2^/g) provided by Meta, CJSC. As a rule, the plant compatible with the thermodesorption method (TDM) consisted of a gas preparation part (usually tanks filled with N_2_- or Ar-adsorbent gases and He carrier gas), an adsorber, a U-shaped tube, and a gas meter. Adsorbent training happened inside the adsorber, which was placed inside a furnace at a temperature range of 200–350 °C and with He or an Ar/He mixture flowing through. After that, the adsorber was cooled to room temperature with the gas still flowing (at such a temperature, Ar and He normally do not adsorb); then, the adsorber was placed inside a cryogenic storage drawer filled with liquid nitrogen—at this temperature, Ar was adsorbed, but the carrier gas (He) could practically still not be adsorbed). After equilibrium saturation at a fixed concentration of Ar, the adsorber was heated to room temperature, the Ar adsorbed at 77 K was released and its quantity was measured with a thermal conductivity detector–katharometer.

The xerogel films of the porous nanocomposites of two- and three-component systems (tin dioxide–silica dioxide and tin dioxide–indium oxide–silica dioxide) were formed through the sol–gel method as a result of spinodal decomposition during the application of polymeric sol to the surface of the base and the thermal treatment of structures, during which metal oxides were formed from nonorganic salts, volatile components were actively released (which led to a significant decrease in mass), gels turned into xerogels (thin films) and porous nanomaterials formed under the conditions of spinodal decomposition.

The xerogel films were created from sols. The sols were applied to all substrates using a centrifuge (3000 revolutions per minute) for 3–5 s. The compositions (concentrations) of nanocomposites were chosen as 10SiO_2_–90SnO_2_, 10SiO_2_–85SnO_2_–5In_2_O_3,_ 10SiO_2_–80SnO_2_–10In_2_O_3_ and 10SiO_2_–75SnO_2_–15In_2_O_3_ (mas. %).

Prepared polymer sol solutions were poured onto the surface of substrates pretreated in acetone, isopropyl alcohol and deionized water, and then dispersed using a centrifuge. During centrifugation, a more complete hydrolysis of alkoxides occurred on the substrate surface in a thin sol layer, followed by polycondensation and the formation of spatial structures in the form of gels. Freshly obtained gels are jelly-like products whose inorganic mesh structure retains water, organic solvents and nonreacting substances. During the heat treatment of gels, the following processes occurred: the formation of metal oxides from nonorganic salts; the intensive release of volatile components accompanied by significant weight loss; the compaction of films; improved adhesion of films to the substrate surface; increase in the mechanical and chemical strength of films; the transition of gels into xerogels.

A study of the morphology of film nanostructures synthesized from sols was carried out using semicontact atomic force microscopy (AFM) with the help of the Ntegra Terma nanolaboratory (NT-MDT, Zelenograd, Russia). To test the surface of the produced samples, NSG 01-type probe sensors (resonance frequency of 150 kHz) were used.

The measurement of the gas sensitivity of the film nanocomposite materials produced using the sol–gel method was conducted through measuring the detection temperature and the resistance of sensory nanostructures in an air atmosphere and in the presence of a reagent gas. The two-probe method was used to measure the resistance. The reagent gas concentration was constant. The measurement of the gas sensitivity was performed in stages, first by heating the sensory nanocomposite in a fixed flow of atmospheric air to the required temperature (300–400 °C), measuring the resistance of the active layer at this temperature, and then sending an impulse of the studied material (concentration 1000 ppm) until the resistance of the active layer in the vapours of this material stabilized. The reduction time period was measured from the moment the source of the reagent gas was turned off till the moment when the layer resistance came back to the initial value ±10%. The measurement of gas sensitivity was determined using reductional fumes of acetone and ethanol; the sensitivity was measured using the following formula: S = (R_AIR_ − R_GAS_)/R_GAS_, where R_AIR_ is the resistance of the sample in the air and R_GAS_ is its resistance in the presence of a reagent gas.

To understand some of the physical–chemical processes that happen during the adsorption of gas molecules on the surface of the produced nanostructures, two models—the Langmuir model and the Brunauer–Emmett–Teller (BET) theory—were used to carry out calculations in the LabVIEW environment.

We compared the results of a phase analysis of the interaction between components during the formation of nanostructures through the use of X-ray diffraction, the method of partial pressure diagrams in a wide range of temperatures and pressures in a computer mathematics system, Mathcad, and reviewing the results of the measurement of nanocomposite sensitivity.

## Figures and Tables

**Table 1:** Temperature dependency lg P_SnO_ = f(T).

T,K	400	505	1000	1250
−lg P_SnO_	42.165	31.345	11.153	7.284

**Table 2:** Phase equilibriums in Sn–Si–O system.

Coexisting Phases	Reactions
Si, SiO_2_	Si_(s)_ + O_2(g)_ = SiO_2(s)_
SiO_2_, SnSiO_3_	SiO_2(s)_ + SnO_(g)_ = SnSiO_3(s)_
Si, SnSiO_3_	Si_(s)_ + O_2(g)_ + SnO_(g)_ = SnSiO_3(s)_

**Table 3:** Temperature dependency lg P_In_ = f(T).

T,K	373	573	1000	1500
−lg P_In_	47.58	28.58	13.073	6.466

**Table 4:** Phase equilibriums in In–Si–O system.

Coexisting Phases	Reactions
Si, SiO_2_	Si_(s)_ + O_2(g)_ = SiO_2(s)_
Si, In_2_(SiO_3_)_3_	3Si_(s)_ + 2In_(g)_ + 9/2O_2(g)_ = In_2_(SiO_3_)_3(s)_
SiO_2_, In_2_(SiO_3_)_3_	3SiO_2(s)_ + 2In_(g)_ + 3/2O_2(g)_ = In_2_(SiO_3_)_3(s)_

**Table 5:** Phase equilibriums in In–Sn–O system.

Coexisting Phases	Reactions
Sn, SnO_2_	Sn_(s)_ + O_2(g)_ = SnO_2(s)_
In, In_2_O_3_	2In_(s)_ + 3/2O_2(g)_ = In_2_O_3(s)_

**Table 6:** Results of calculations of equilibrium reaction constants.

Subsystem, Reaction and Equilibrium Equation Line	T, K	lgK_p_
Sn-Si-O: Si_(s)_ + O_2(g)_ = SiO_2(s)_ lgP_O2_ = −lgK_p_	873 1073	41.365 31.872
SiO_2(s)_ + SnO_(g)_ = SnSiO_3(s)_ lgP_SnO_ = −lgK_p_	873 1073	9.385 5.503
Si_(s)_ + O_2(g)_ + SnO_(g)_ = SnSiO_3(s)_ lgP_SnO_ = −lgP_O2_ − lgK_p_	873 1073	50.738 37.365
In-Si-O: Si_(s)_ + O_2(g)_ = SiO_2(s)_ lgP_O2_ = –lgK_p_	873 1073	41.365 31.872
3Si_(s)_ + 2In_(g)_ + 9/2O_2(g)_ = In_2_(SiO_3_)_3(s)_ 2lgP_In_ = −9/2 lgP_O2_ − lgK_p_	873 1073	66.497 41.549
3SiO_2(s)_ + 2In_(g)_ + 3/2O_2(g)_ = In_2_(SiO_3_)_3(s)_ 2lgP_In_ = −3/2 lgP_O 2_− lgK_p_	873 1073	−57.603 −54.103
In-Sn-O: Sn_(s)_ + O_2(g)_ = SnO_2(s)_ lgP_O2_ = −lgK_p_	873 1073	24.025 17.526
2In_(s)_ + 3/2O_2(g)_ = In_2_O_3(s)_ lgP_O2_ = −2/3 lgK_p_	873 1073	38.791 28.434
Calculations of pressures of saturated vapours		
Sn-Si-O, In-Sn-O: SnO_(s)_ = SnO_(g)_ LgP_SnO_ = lgK_p_	873 1073	−8.254 −4.503
In-Si-O, In-Sn-O: In_(s)_ = In_(g)_ LgP_In_ = lgK_p_	873 1073	−8.637 −5.965

## Data Availability

Not applicable.
